# Genome Sequences of Anelloviruses, Genomovirus, and Papillomavirus Isolated from Nasal Pharyngeal Swabs

**DOI:** 10.1128/mra.00681-22

**Published:** 2022-08-16

**Authors:** Courtney L. Collins, Simona Kraberger, Rafaela S. Fontenele, Temitope O. C. Faleye, Deborah Adams, Sangeet Adhikari, Helen Sandrolini, Sarah Finnerty, Rolf U. Halden, Matthew Scotch, Arvind Varsani

**Affiliations:** a The Biodesign Center for Fundamental and Applied Microbiomics, School of Life Sciences, Center for Evolution and Medicine, Arizona State University, Tempe, AZ, USA; b Biodesign Center for Environmental Health Engineering, Biodesign Institute, Arizona State University, Tempe, AZ, USA; c Biodesign Center for Personalized Diagnostics, Biodesign Institute, Arizona State University, Tempe, AZ, USA; d School of Sustainable Engineering and the Built Environment, Arizona State University, Tempe, AZ, USA; e Arizona State University Health Services, Arizona State University, Tempe, AZ, USA; f College of Health Solutions, Arizona State University, Phoenix, AZ, USA; DOE Joint Genome Institute

## Abstract

The genome sequences of three anelloviruses (genus *Alphatorquevirus*), a genomovirus (genus *Gemykolovirus*), and an unclassified papillomavirus were identified in four human nasopharyngeal swabs, and one was positive for influenza A and one for influenza B virus. The influenza B virus-positive sample had a coinfection with an anellovirus and a papillomavirus.

## ANNOUNCEMENT

**FIG 1:**
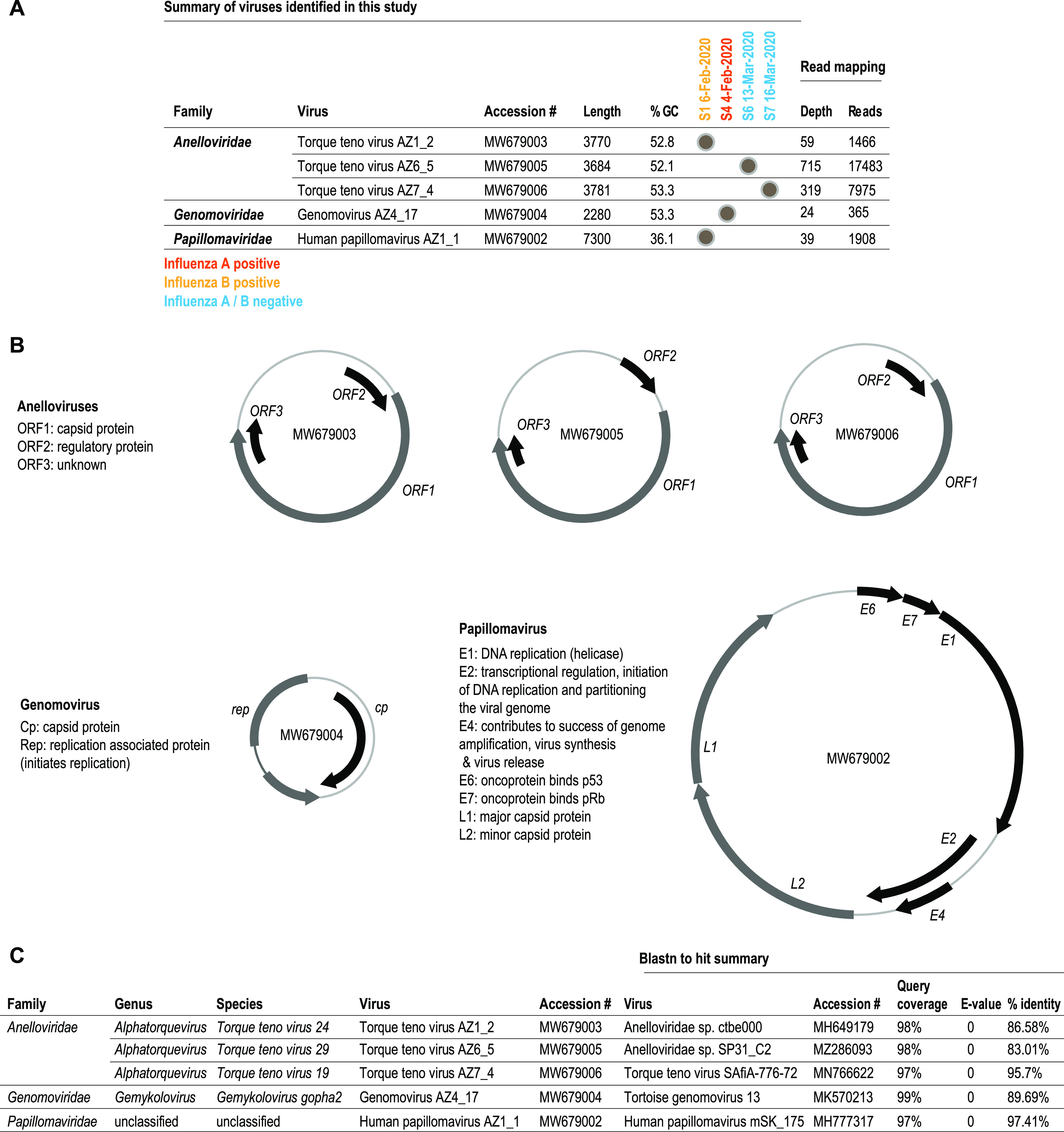
(A) Summary of the genomes determined from the four nasopharyngeal swabs with accession number, genome length, GC content, read depth, and coverage. (B) Genome organization of the anelloviruses, genomovirus, and papillomavirus identified from the swab samples. (C) Taxonomic classification of the anelloviruses, genomovirus, and papillomavirus and the summary of the BLASTn analysis.

Nasal pharyngeal swabs were taken from four patients with influenza-like illness as part of a routine clinical testing for influenza virus on a university campus in Arizona between February and March 2020. Two of these swabs tested positive for seasonal influenza viruses, namely, one influenza A virus (IAV) and the other influenza B virus (IBV), via rapid lateral flow immunoassay (Abbott BinaxNow). To identify associated DNA viruses in these samples, 200 μL of the buffer from the lateral flow assay was used to extract viral DNA with the high pure viral nucleic acid kit (Roche Diagnostics, USA). Circular DNA was amplified by rolling circle amplification (RCA) using an Illustra TempliPhi kit (GE Healthcare, USA). The RCA products were used to prepare libraries using a TruSeq Nano DNA kit (Illumina, USA). The 2× 150-bp libraries were sequenced on a NovaSeq6000 sequencer at Psomagen Inc. (USA). The reads were trimmed using Trimmomatic v0.39 (1) and *de novo* assembled with metaSPAdes 3.14.0 (2). We identified complete genome sequences (based on terminal redundancy) of anelloviruses (*n* = 3), genomovirus (*n* = 1), and a papillomavirus (*n* = 1) using the BLASTn (3) nonredundant (nr) database online on May 2021. Reads were mapped to the viral genome sequences using BBMap (4) and showed depth coverage of 24× to 715× and mapped reads per genome of 365 to 17,483 (Fig. 1A). In sample S1 (IBV positive), we found a coinfection with an anellovirus and a papillomavirus (Fig. 1).

Anelloviruses are circular single-stranded DNA (ssDNA) viruses (2.0 to 3.9 kb) (5). They are diverse and highly prevalent but are not associated with disease (6). Anellovirus genomes have three large open reading frames (ORFs). The three anelloviruses identified here are ~3.7 kb (with GC content of 52% to 53%) and share >83% nucleotide identity with those previously identified in humans (Fig. 1B and C). Based on the guidelines for the classification of mammal-infecting members of the *Anelloviridae* family (7), torque teno virus AZ1_2 (MW679003), torque teno virus AZ6_5 (MW679005), and torque teno virus AZ7_4 (MW679006) identified in the nasal pharyngeal swabs all belong to the genus *Alphatorquevirus* and species *Torque teno virus 24*, *Torque teno virus 29*, and *Torque teno virus 19*, respectively (Fig. 1C).

Genomoviruses are circular ssDNA viruses (2 to 2.4 kb) that encode bidirectionally transcribed ORFs, capsid protein (CP), and a replication associated protein (Rep), with the exception of Fusarium graminearum gemytripvirus 1 which is a tripartite virus (8–10). Although ubiquitous in nature, only two genomoviruses have confirmed hosts, namely, Sclerotinia sclerotiorum (11) and Fusarium graminearum (12). Genomovirus AZ4_17 (GenBank accession number MW679004; 2.28 kb with GC content of 53.3%) shares ~89.6% genome-wide nucleotide identity with tortoise genomovirus 13 (MK570213) (Fig. 1C) (13) and is part of the genus *Gemykolovirus* and species *Gemykolovirus gopha2* based on the classification guideline for *Genomoviridae* (10). Genomoviruses have been identified previously in human blood, cerebrospinal fluid, and pericardial fluid (14–18), and we speculate that the one identified here may be those that infect oral fungi (yeast).

Papillomaviruses are circular double-stranded DNA viruses (~5.7 to 8.6 kb) and are classified within two subfamilies (*Firstpapillomavirinae* and *Secondpapillomavirinae*) (19). Members of the *Firstpapillomavirinae* encode at least seven genes (19). Human papillomavirus AZ1_1 (MW679002; 7.3 kb with GC content of 36.1%) identified in the swab shares ~97% nucleotide identity with HPV-mSK_175 (MH777317) from a human skin swab (20) (Fig. 1) and HPV_SD2R (KC113191) from an oral swab (21); neither have been assigned a type or classified.

Sample collection was part of routine clinical care which is approved by Arizona State University Institutional Review under study identifier (ID) number STUDY00008985.

### Data availability.

The sequences of the viruses identified in this study have been deposited in GenBank with accession numbers MW679002, MW679003, MW679004, MW679005, and MW679006. Raw reads have been deposited in SRA project number PRJNA701833 with SRA accessions SRR13720055, SRR13720056, SRR13720057, and SRR13720061.

## References

[1] Bolger AM, Lohse M, Usadel B. 2014. Trimmomatic: a flexible trimmer for Illumina sequence data. Bioinformatics 30:2114–2120. doi:10.1093/bioinformatics/btu170. 24695404 PMC4103590

[2] Bankevich A, Nurk S, Antipov D, Gurevich AA, Dvorkin M, Kulikov AS, Lesin VM, Nikolenko SI, Pham S, Prjibelski AD, Pyshkin AV, Sirotkin AV, Vyahhi N, Tesler G, Alekseyev MA, Pevzner PA. 2012. SPAdes: a new genome assembly algorithm and its applications to single-cell sequencing. J Comput Biol 19:455–477. doi:10.1089/cmb.2012.0021. 22506599 PMC3342519

[3] Altschul SF, Gish W, Miller W, Myers EW, Lipman DJ. 1990. Basic local alignment search tool. J Mol Biol 215:403–410. doi:10.1016/S0022-2836(05)80360-2. 2231712

[4] Bushnell B. 2014. BBMap: a fast, accurate, splice-aware aligner. Lawrence Berkeley National Lab., Berkeley, CA.

[5] Biagini P, Bendinelli M, Hino S, Kakkola L, Mankertz A, Niel C, Okamoto H, Raidal S, Teo CG, Todd D. 2012. Family Anelloviridae, pp 331–341. *In* King AMQ, Adams EB, Carstens EB, Lefkowitz EJ. (ed) Virus taxonomy: ninth report of the International Committee on Taxonomy of Viruses. Academic Press, Waltham, MA.

[6] Freer G, Maggi F, Pifferi M, Di Cicco ME, Peroni DG, Pistello M. 2018. The virome and its major component, anellovirus, a convoluted system molding human immune defenses and possibly affecting the development of asthma and respiratory diseases in childhood. Front Microbiol 9:686. doi:10.3389/fmicb.2018.00686. 29692764 PMC5902699

[7] Varsani A, Opriessnig T, Celer V, Maggi F, Okamoto H, Blomstrom AL, Cadar D, Harrach B, Biagini P, Kraberger S. 2021. Taxonomic update for mammalian anelloviruses (family Anelloviridae). Arch Virol 166:2943–2953. doi:10.1007/s00705-021-05192-x. 34383165

[8] Krupovic M, Ghabrial SA, Jiang D, Varsani A. 2016. Genomoviridae: a new family of widespread single-stranded DNA viruses. Arch Virol 161:2633–2643. doi:10.1007/s00705-016-2943-3. 27343045

[9] Varsani A, Krupovic M. 2017. Sequence-based taxonomic framework for the classification of uncultured single-stranded DNA viruses of the family Genomoviridae. Virus Evol 3:vew037. doi:10.1093/ve/vew037. 28458911 PMC5399927

[10] Varsani A, Krupovic M. 2021. Family Genomoviridae: 2021 taxonomy update. Arch Virol 166:2911–2926. doi:10.1007/s00705-021-05183-y. 34331585

[11] Yu X, Li B, Fu Y, Jiang D, Ghabrial SA, Li G, Peng Y, Xie J, Cheng J, Huang J, Yi X. 2010. A geminivirus-related DNA mycovirus that confers hypovirulence to a plant pathogenic fungus. Proc Natl Acad Sci USA 107:8387–8392. doi:10.1073/pnas.0913535107. 20404139 PMC2889581

[12] Li P, Wang S, Zhang L, Qiu D, Zhou X, Guo L. 2020. A tripartite ssDNA mycovirus from a plant pathogenic fungus is infectious as cloned DNA and purified virions. Sci Adv 6:eaay9634. doi:10.1126/sciadv.aay9634. 32284975 PMC7138691

[13] Orton JP, Morales M, Fontenele RS, Schmidlin K, Kraberger S, Leavitt DJ, Webster TH, Wilson MA, Kusumi K, Dolby GA, Varsani A. 2020. Virus discovery in desert tortoise fecal samples: novel circular single-stranded DNA viruses. Viruses 12:143. doi:10.3390/v12020143. PMC7077246 31991902

[14] Halary S, Duraisamy R, Fancello L, Monteil-Bouchard S, Jardot P, Biagini P, Gouriet F, Raoult D, Desnues C. 2016. Novel single-stranded DNA circular viruses in pericardial fluid of patient with recurrent pericarditis. Emerg Infect Dis 22:1839–1841. doi:10.3201/eid2210.160052. 27648613 PMC5038422

[15] Lamberto I, Gunst K, Muller H, Zur Hausen H, de Villiers EM. 2014. Mycovirus-like DNA virus sequences from cattle serum and human brain and serum samples from multiple sclerosis patients. Genome Announc 2:e00848-14. doi:10.1128/genomeA.00848-14. 25169858 PMC4148726

[16] Uch R, Fournier PE, Robert C, Blanc-Tailleur C, Galicher V, Barre R, Jordier F, de Micco P, Raoult D, Biagini P. 2015. Divergent Gemycircularvirus in HIV-positive blood, France. Emerg Infect Dis 21:2096–2098. doi:10.3201/eid2111.150486. 26488181 PMC4622245

[17] Zhang W, Li L, Deng X, Blumel J, Nubling CM, Hunfeld A, Baylis SA, Delwart E. 2016. Viral nucleic acids in human plasma pools. Transfusion 56:2248–2255. doi:10.1111/trf.13692. 27306718

[18] Zhou C, Zhang S, Gong Q, Hao A. 2015. A novel gemycircularvirus in an unexplained case of child encephalitis. Virol J 12:197. doi:10.1186/s12985-015-0431-0. 26596706 PMC4657213

[19] Van Doorslaer K, Chen Z, Bernard HU, Chan PKS, DeSalle R, Dillner J, Forslund O, Haga T, McBride AA, Villa LL, Burk RD, ICTV Report Consortium. 2018. ICTV virus taxonomy profile: Papillomaviridae. J Gen Virol 99:989–990. doi:10.1099/jgv.0.001105. 29927370 PMC6171710

[20] Tirosh O, Conlan S, Deming C, Lee-Lin SQ, Huang X, Program NCS, Su HC, Freeman AF, Segre JA, Kong HH, NISC Comparative Sequencing Program. 2018. Expanded skin virome in DOCK8-deficient patients. Nat Med 24:1815–1821. doi:10.1038/s41591-018-0211-7. 30397357 PMC6286253

[21] Mokili JL, Dutilh BE, Lim YW, Schneider BS, Taylor T, Haynes MR, Metzgar D, Myers CA, Blair PJ, Nosrat B, Wolfe ND, Rohwer F. 2013. Identification of a novel human papillomavirus by metagenomic analysis of samples from patients with febrile respiratory illness. PLoS One 8:e58404. doi:10.1371/journal.pone.0058404. 23554892 PMC3600855

